# Development and Internal Validation of a Nomogram for Identifying Postpartum Fatigue in Postpartum Women: A Cross‐Sectional Study

**DOI:** 10.1002/nop2.70907

**Published:** 2026-10-09

**Authors:** Zhidong Guo, Min Xu, Shouzhen Cheng

**Affiliations:** ^1^ Department of Obstetrics and Gynecology The First Affiliated Hospital of Sun Yat‐sen University Guangzhou People's Republic of China; ^2^ Nursing Department The First Affiliated Hospital of Sun Yat‐sen University Guangzhou People's Republic of China

## Abstract

**Aim:**

To identify factors associated with postpartum fatigue in postpartum women and to develop and internally validate a clinical nomogram.

**Design:**

A cross‐sectional study.

**Methods:**

This cross‐sectional study recruited 780 postpartum women via convenience sampling at a tertiary hospital in Guangdong, China (January–May 2025). After excluding invalid questionnaires, 744 participants were randomly allocated (7:3) to derivation and internal validation sets. Data were collected using a general information questionnaire and validated scales for postpartum fatigue, depression and social support. Candidate factors were pre‐specified based on clinical relevance and previous evidence. LASSO regression was used for variable selection. Model performance was assessed using the AUC, calibration plots, the Hosmer‐Lemeshow test and decision curve analysis (DCA), with internal validation by randomly splitting the sample into derivation and internal validation sets.

**Results:**

Among the 744 postpartum women, the prevalence of postpartum fatigue was 69.0% (*n* = 513). Four independent factors were associated with postpartum fatigue: postpartum depression, maternal sleep quality, breastfeeding ability and intrapartum blood loss. The nomogram showed good discrimination in both the derivation (AUC = 0.821) and internal validation (AUC = 0.801) sets. At the optimal cut‐off of 0.682, sensitivity and specificity were 75.2% and 76.8%, respectively. The Hosmer‐Lemeshow test confirmed good calibration in both sets (derivation: *χ* ^2^ = 8.949, *p* = 0.3466; internal validation: *χ* ^2^ = 4.422, *p* = 0.8172). DCA indicated favourable clinical net benefit across a relevant range of thresholds.

**Conclusions:**

We developed and internally validated a practical nomogram for identifying postpartum fatigue using readily available clinical and psychosocial factors. The model showed good performance.

**Implications for Nursing Practice:**

This nomogram may assist nurses and midwives in early identification of postpartum women at increased likelihood of fatigue and facilitate individualized nursing interventions to improve postpartum recovery.

**Patient or Public Contribution:**

No patient or public contribution was made to the design, conduct, analysis, interpretation or writing of this study.

## 1 Introduction

Postpartum fatigue is defined as a state of overwhelming exhaustion after childbirth that reduces a woman's physical and mental capacity and is not relieved by rest (Baattaiah et al. 2024; Sultan and Carvalho 2021). Regarded as one of the most frequent complications of the puerperium, more than half of new mothers suffer from it (Zhuang et al. 2024). This condition causes difficulties that go beyond general tiredness and may impair functioning in different aspects of early motherhood. This has been shown to affect maternal recovery, success in breastfeeding, competence in infant care, mother‐baby bonding and family adjustment (Balshi and Bove 2024; Baattaiah et al. 2023; Lunsford‐Avery et al. 2026; Khamidullina et al. 2025).

For a large number of women, this powerful exhaustion does not wear off during the early postpartum period. Moreover, it may persist and affect women's long‐term health and well‐being. Despite its high prevalence and adverse impact, postpartum fatigue remains under‐recognized in routine postpartum care. Many clinicians and new mothers consider this to be a normal part of the puerperium. This misconception poses a major barrier to care. As a result, postpartum fatigue is often insufficiently screened and managed in clinical practice. To address this important gap in postpartum care, timely identification of women with postpartum fatigue must be made a clinical priority. Therefore, this study aimed to develop and internally validate a model for the concurrent identification of postpartum fatigue at 6 weeks postpartum. The target population was women attending routine postpartum follow‐up, and the nomogram was intended to support obstetric clinicians in identifying women at high probability of postpartum fatigue during the postpartum care pathway.

## 2 Methods

### 2.1 Study Design and Participants

Between January to May 2025, a cross‐sectional study using convenience sampling recruited women attending their normal six‐week postpartum visit at the obstetrics outpatient clinic of a tertiary hospital in Guangdong, China. Women were eligible if they were: (1) aged ≥ 20 years; (2) assessed between 42 ± 7 days postpartum; (3) recovering from a singleton live birth; (4) capable of independently completing the study questionnaires; and (5) willing to provide written informed consent to participate in the study. Women were excluded if they had (1) significant mother‐infant separation that prevented the mother from being the primary caregiver; (2) a previous diagnosis of severe medical or psychiatric disease; or (3) major postpartum complications, such as eclampsia or severe postpartum haemorrhage requiring intensive care. To ensure ethical compliance, ethical approval was obtained from the institutional ethics committee (Approval No. [2024]042) and written informed consent was obtained from all subjects prior to enrolment.

### 2.2 Sample Size

The sample size was determined according to the events‐per‐variable (EPV) principle, requiring at least 10 outcome events per factor. With 25 candidate factors and an anticipated postpartum fatigue prevalence of 58% (Zhuang et al. 2024), at least 431 participants were required to obtain 250 outcome events. After accounting for a 15% loss rate, the minimum required sample size was 507 participants. To ensure adequate power for model development and internal validation, 780 women were ultimately recruited.

### 2.3 Instruments

Maternal characteristics and psychological status were assessed using a self‐designed general information questionnaire and validated scales. Sociodemographic data, infant‐feeding practices and infant‐care practices were collected using the general information questionnaire developed for this study. Information on delivery mode, time of delivery, intrapartum blood loss and labour duration was obtained from medical records. Sociodemographic variables included age, educational level, household income, occupation and residence. Obstetric variables included parity, gestational age, mode of delivery, labour duration and intrapartum blood loss.

Postpartum fatigue was assessed using the Postpartum Fatigue Scale (PFS), a 14‐item validated instrument widely used in the Chinese population (Wang and Xue 2007). The PFS includes three dimensions: physical fatigue, mental fatigue and consequences of fatigue. Each item is rated on a 5‐point Likert scale. Higher scores correspond to greater fatigue severity, with higher standardized scores indicating greater fatigue severity. Based on the total scores, fatigue was categorized into four levels: severe (81–100 points), moderate (41–80 points), mild (3–40 points) and normal (below 3 points). In this study, a PFS standardized score > 40 was defined as postpartum fatigue, whereas a score ≤ 40 was considered non‐fatigue. The scale demonstrated excellent internal consistency in our sample (Cronbach's *α* = 0.95).

Postpartum depressive symptoms were measured using the 10‐item Edinburgh Postnatal Depression Scale (EPDS), which has been validated in Chinese populations (Huang and Kong 2022). Each item is scored on a 4‐point scale from 0 to 3, yielding a total score ranging from 0 to 30. Higher scores indicate more severe depressive symptoms. In this study, the Chinese validated version was used and the scale showed good internal consistency (Cronbach's *α* = 0.86).

Perceived social support was measured using the 12‐item Perceived Social Support Scale (PSSS), validated in the Chinese population (Zhong et al. 2005). The PSSS uses a 7‐point Likert scale, with each item scored from 1 to 7. Total scores range from 12 to 84, with higher scores indicating greater perceived social support. The scale demonstrated acceptable internal consistency in this study (Cronbach's *α* = 0.90).

### 2.4 Data Collection

To minimize bias, all data collectors received standardized training. After having undergone standardized training, the data collectors systematically informed eligible participants about the study's purpose, the study procedures and secured their written informed consent. Data were collected using an electronic questionnaire on the Wenjuanxing platform, which participants accessed via a QR code on their mobile phones. In line with the principle of data integrity, the system was set to block duplicate form submissions from the same device. Also, all the fields were made compulsory. Questionnaires with missing key variables were excluded from analysis. In the later stages of data cleaning, we removed submissions with patterned responses or unrealistically short completion times.

### 2.5 Statistical Analysis

The statistical analyses were performed using SPSS version 24.0 (IBM Corp., Armonk, NY, USA) and R version 4.4.3 (R Foundation for Statistical Computing, Vienna, Austria). Prior to modelling, the full sample was randomly divided into a derivation set (70%) and a internal validation set (30%).

There were no missing data for the variables included in the final analysis because all questionnaire items were mandatory in the online survey platform. Categorical nominal variables were dummy coded using reference categories. Ordinal variables were treated as categorical variables unless a linear relationship with the outcome was confirmed. Continuous variables were entered into the multivariable logistic regression model using their original measured values, without categorization. Descriptive statistics were first calculated to summarise the participant characteristics. Chi‐square tests were used to compare categorical variables with sufficient expected cell counts, whereas Fisher's exact test was employed for categorical variables with sparse distributions, including forceps delivery and self‐care. Independent *t*‐tests or Mann–Whitney *U* tests were used for continuous variables, as appropriate, to explore preliminary associations with postpartum fatigue. Multicollinearity among factors was assessed using variance inflation factors (VIFs), with VIF > 5 indicating potential collinearity. Candidate associated factors were selected a priori based on clinical relevance, previous literature and feasibility in routine postpartum assessment. Before model construction, multicollinearity among candidate factors was assessed. LASSO regression with 10‐fold cross‐validation was then applied in the derivation set to identify the most informative factors for inclusion in the multivariable logistic regression model. The final model was developed using the selected factors, and a nomogram was constructed based on the final model. The PFS standardised score was dichotomised at > 40 to define postpartum fatigue. Other scale scores were initially treated as continuous factors in the univariable and multivariable analyses.

Model performance was evaluated in both the derivation and internal validation sets. Discrimination was assessed using the area under the receiver operating characteristic curve (AUC), calibration was assessed using calibration plots and the Hosmer–Lemeshow test. Clinical utility was assessed using decision curve analysis (DCA).

Internal validation was conducted using a randomly split validation set. The optimal cutoff value was determined by the maximum Youden index.

## 3 Results

### 3.1 Participant Characteristics

Of the 780 eligible participants surveyed between January 2025 and May 2025, 36 questionnaires were excluded because of extremely short completion times and highly consistent responses. Ultimately, 744 questionnaires were deemed eligible for analysis, corresponding to a response rate of 95.4%. These participants were randomly divided into a derivation set (*n* = 522) and a internal validation set (*n* = 222) (Figure 1). The mean age of the participants was 30.6 ± 4.2 years; most participants holding a bachelor's degree or higher (90.7%); and mainly were primiparous (64.3%). No significant differences were observed between the two sets in baseline sociodemographic or obstetric characteristics, indicating that the random allocation was successful (all *p* > 0.05; Table 1).

**FIGURE 1:**
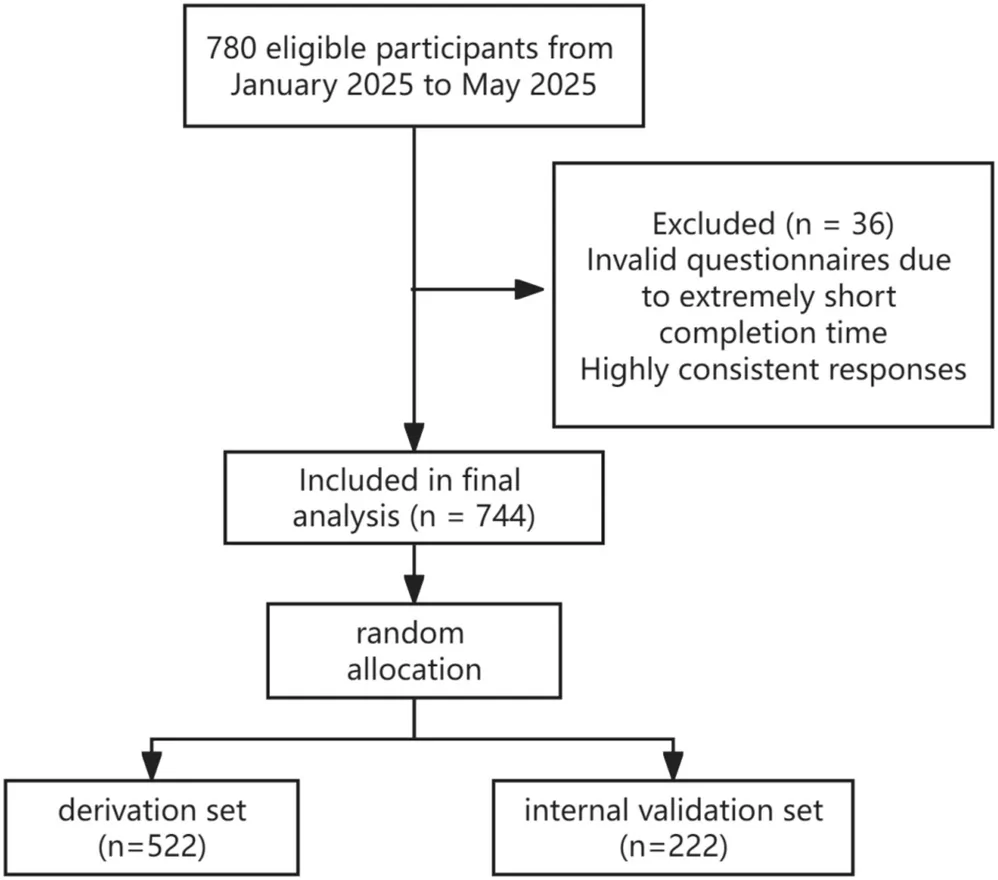
Participants flow diagram.

**TABLE 1:** Comparison of general characteristics between derivation set and internal validation set (*n* = 744).

Characteristic	All	Internal validation set	Derivation set	*p*
	*n* = 744	*n* = 222	*n* = 522	
Age group (years)				0.800^b^
< 35	609 (81.85%)	180 (81.08%)	429 (82.18%)	
≥ 35	135 (18.15%)	42 (18.92%)	93 (17.82%)	
Education level				0.411^b^
High school or below	69 (9.27%)	16 (7.21%)	53 (10.15%)	
Bachelor's degree	595 (79.97%)	180 (81.08%)	415 (79.50%)	
Postgraduate degree	80 (10.75%)	26 (11.71%)	54 (10.34%)	
Occupation				0.455^b^
Farmer	54 (7.26%)	21 (9.46%)	33 (6.32%)	
Employee	328 (44.09%)	93 (41.89%)	235 (45.02%)	
Civil servant	192 (25.81%)	59 (26.58%)	133 (25.48%)	
Self‐employed	170 (22.85%)	49 (22.07%)	121 (23.18%)	
Monthly household income (RMB)				0.653^b^
–5999	173 (23.25%)	48 (21.62%)	125 (23.95%)	
6000–7999	134 (18.01%)	38 (17.12%)	96 (18.39%)	
8000–9999	101 (13.58%)	35 (15.77%)	66 (12.64%)	
10,000—	336 (45.16%)	101 (45.50%)	235 (45.02%)	
Mode of delivery				0.684^c^
Vaginal delivery	332 (44.62%)	96 (43.24%)	236 (45.21%)	
Forceps delivery	32 (4.30%)	8 (3.60%)	24 (4.60%)	
Caesarean section	380 (51.08%)	118 (53.15%)	262 (50.19%)	
Gestational age (weeks)				0.402^b^
< 37	58 (7.80%)	14 (6.31%)	44 (8.43%)	
≥ 37	686 (92.20%)	208 (93.69%)	478 (91.57%)	
Parity				0.391^b^
Primiparous	478 (64.25%)	137 (61.71%)	341 (65.33%)	
Multiparous	266 (35.75%)	85 (38.29%)	181 (34.67%)	
Intrapartum blood loss (mL)	241.14 ± 88.09	239.55 ± 87.71	241.82 ± 88.32	0.747^a^
PSSS	68.33 ± 8.80	68.14 ± 8.93	68.41 ± 8.76	0.703^a^
Family support	24.12 ± 3.39	24.05 ± 3.39	24.16 ± 3.39	0.675^a^
Friend support	22.87 ± 3.31	22.82 ± 3.36	22.90 ± 3.29	0.761^a^
Other support	21.33 ± 3.16	21.27 ± 3.31	21.35 ± 3.09	0.771^a^
EPDS	7.05 ± 4.06	7.01 ± 4.13	7.06 ± 4.03	0.889^a^
Outcome				1.000^b^
Non‐fatigue	231 (31.05%)	69 (31.08%)	162 (31.03%)	
Fatigue	513 (68.95%)	153 (68.92%)	360 (68.97%)	
Primary caregiver				0.774^c^
Maternity matron	303 (40.73%)	89 (40.09%)	214 (41.00%)	
Family member	425 (57.12%)	127 (57.21%)	298 (57.09%)	
Self‐care	16 (2.15%)	6 (2.70%)	10 (1.92%)	
Maternal sleep quality				0.085^b^
Very satisfied	85 (11.42%)	17 (7.66%)	68 (13.03%)	
Satisfied	219 (29.44%)	68 (30.63%)	151 (28.93%)	
Fair	367 (49.33%)	109 (49.10%)	258 (49.43%)	
Dissatisfied	73 (9.81%)	28 (12.61%)	45 (8.62%)	
Infant care ability				0.059^c^
Very satisfied	84 (11.29%)	15 (6.76%)	69 (13.22%)	
Satisfied	280 (37.63%)	90 (40.54%)	190 (36.40%)	
Fair	343 (46.10%)	108 (48.65%)	235 (45.02%)	
Dissatisfied	37 (4.97%)	9 (4.05%)	28 (5.36%)	
Breastfeeding ability				0.907^b^
Proficient	389 (52.28%)	117 (52.70%)	272 (52.11%)	
Partially proficient	192 (25.81%)	55 (24.77%)	137 (26.25%)	
Non‐proficient	163 (21.91%)	50 (22.52%)	113 (21.65%)	
Infant feeding method				0.051^b^
Exclusive breastfeeding	200 (26.88%)	49 (22.07%)	151 (28.93%)	
Mixed feeding	373 (50.13%)	126 (56.76%)	247 (47.32%)	
Formula feeding	171 (22.98%)	47 (21.17%)	124 (23.75%)	

*Note:* Data are presented as *n* (%) or mean ± SD.

Abbreviations: EPDS, Edinburgh Postnatal Depression Scale; PSSS, Perceived Social Support Scale.

^a^ *T*‐test.

^b^ Chi‐squared test.

^c^ Fisher's exact test.

### 3.2 Prevalence and Associated Factors of Postpartum Fatigue

The overall prevalence of postpartum fatigue was 69.0% (*n* = 513) in the study population. In the univariate analyses, postpartum fatigue was significantly associated with intrapartum blood loss, maternal sleep quality, breastfeeding ability, perceived social support and postpartum depression score (all *p* < 0.05; Table 2). No significant multicollinearity was detected among the candidate factors (all VIFs < 5).

**TABLE 2:** Comparison of general characteristics between postpartum women with and without fatigue (*n* = 522).

Characteristic	Non‐fatigue group	Fatigue group	*p*
	*N* = 162	*N* = 360	
Age group (years)			0.252^b^
< 35	128 (79.01%)	301 (83.61%)	
≥ 35	34 (20.99%)	59 (16.39%)	
Education level			0.535^b^
High school or below	20 (12.35%)	33 (9.17%)	
Bachelor's degree	126 (77.78%)	289 (80.28%)	
Postgraduate degree	16 (9.88%)	38 (10.56%)	
Occupation			0.479^b^
Farmer	10 (6.17%)	23 (6.39%)	
Employee	81 (50.00%)	154 (42.78%)	
Civil servant	38 (23.46%)	95 (26.39%)	
Self‐employed	33 (20.37%)	88 (24.44%)	
Monthly household income (RMB)			0.322^b^
–5999	34 (20.99%)	91 (25.28%)	
6000–7999	36 (22.22%)	60 (16.67%)	
8000–9999	23 (14.20%)	43 (11.94%)	
10,000—	69 (42.59%)	166 (46.11%)	
Parity			0.208^b^
Primiparous	99 (61.11%)	242 (67.22%)	
Multiparous	63 (38.89%)	118 (32.78%)	
Gestational age (weeks)			0.774^b^
< 37	15 (9.26%)	29 (8.06%)	
≥ 37	147 (90.74%)	331 (91.94%)	
Mode of delivery			0.190^c^
Vaginal delivery	70 (43.21%)	166 (46.11%)	
Forceps delivery	4 (2.47%)	20 (5.56%)	
Caesarean section	88 (54.32%)	174 (48.33%)	
Time of delivery			0.476^b^
22:00–6:00	26 (16.05%)	74 (20.56%)	
6:00–14:00	78 (48.15%)	162 (45.00%)	
14:00–22:00	58 (35.80%)	124 (34.44%)	
Intrapartum blood loss (mL)	225.74 ± 69.69	249.06 ± 94.73	0.002^a^
Postpartum depression (EPDS)	4.62 ± 3.31	8.16 ± 3.84	< 0.001^a^
Perceived social support (PSSS)	70.81 ± 8.50	67.32 ± 8.67	< 0.001^a^
Family support	25.08 ± 3.02	23.74 ± 3.47	< 0.001^a^
Friend support	23.68 ± 3.17	22.54 ± 3.29	< 0.001^a^
Other support	22.06 ± 3.20	21.03 ± 2.99	0.001^a^
Primary caregiver			0.645^c^
Maternity matron	71 (43.83%)	143 (39.72%)	
Family member	88 (54.32%)	210 (58.33%)	
Self‐care	3 (1.85%)	7 (1.94%)	
Maternal sleep quality			< 0.001^c^
Very satisfied	28 (17.28%)	40 (11.11%)	
Satisfied	69 (42.59%)	82 (22.78%)	
Fair	58 (35.80%)	200 (55.56%)	
Dissatisfied	7 (4.32%)	38 (10.56%)	
Infant care ability			< 0.001^c^
Very satisfied	29 (17.90%)	40 (11.11%)	
Satisfied	76 (46.91%)	114 (31.67%)	
Fair	56 (34.57%)	179 (49.72%)	
Dissatisfied	1 (0.62%)	27 (7.50%)	
Breastfeeding ability			< 0.001^b^
Proficient	108 (66.67%)	164 (45.56%)	
Partially proficient	34 (20.99%)	103 (28.61%)	
Non‐proficient	20 (12.35%)	93 (25.83%)	
Infant feeding method			0.046^b^
Exclusive breastfeeding	47 (29.01%)	104 (28.89%)	
Mixed feeding	87 (53.70%)	160 (44.44%)	
Formula feeding	28 (17.28%)	96 (26.67%)	

*Note:* Data are presented as *n* (%) or mean ± SD.

Abbreviations: EPDS, Edinburgh Postnatal Depression Scale; PSSS, Perceived Social Support Scale.

^a^ *T*‐test.

^b^ Chi‐squared test.

^c^ Fisher's exact test.

To avoid overfitting and instability associated with stepwise selection, we employed LASSO regression with 10‐fold cross‐validation to screen the 25 candidate factors. At the optimal *λ*.min, variables with non‐zero coefficients were identified (Figure 2). The variable coding and assignment for the logistic regression analysis are summarized in Table 3. These variables were subsequently entered into a multivariable logistic regression model, which revealed that postpartum depression, maternal sleep quality, breastfeeding proficiency and intrapartum blood loss were independent factors associated with postpartum fatigue (all *p* < 0.05; Table 4). The final logistic regression model was expressed as: logit(*p*) = −3.757 + 0.260 × (postpartum depression) + 0.428 × (maternal sleep quality) + 0.641 × (breastfeeding ability) + 0.350 × (intrapartum blood loss per 100 mL). The probability of postpartum fatigue estimated by the model can be calculated as *p* = 1/(1 + exp.(−logit)).

**FIGURE 2:**
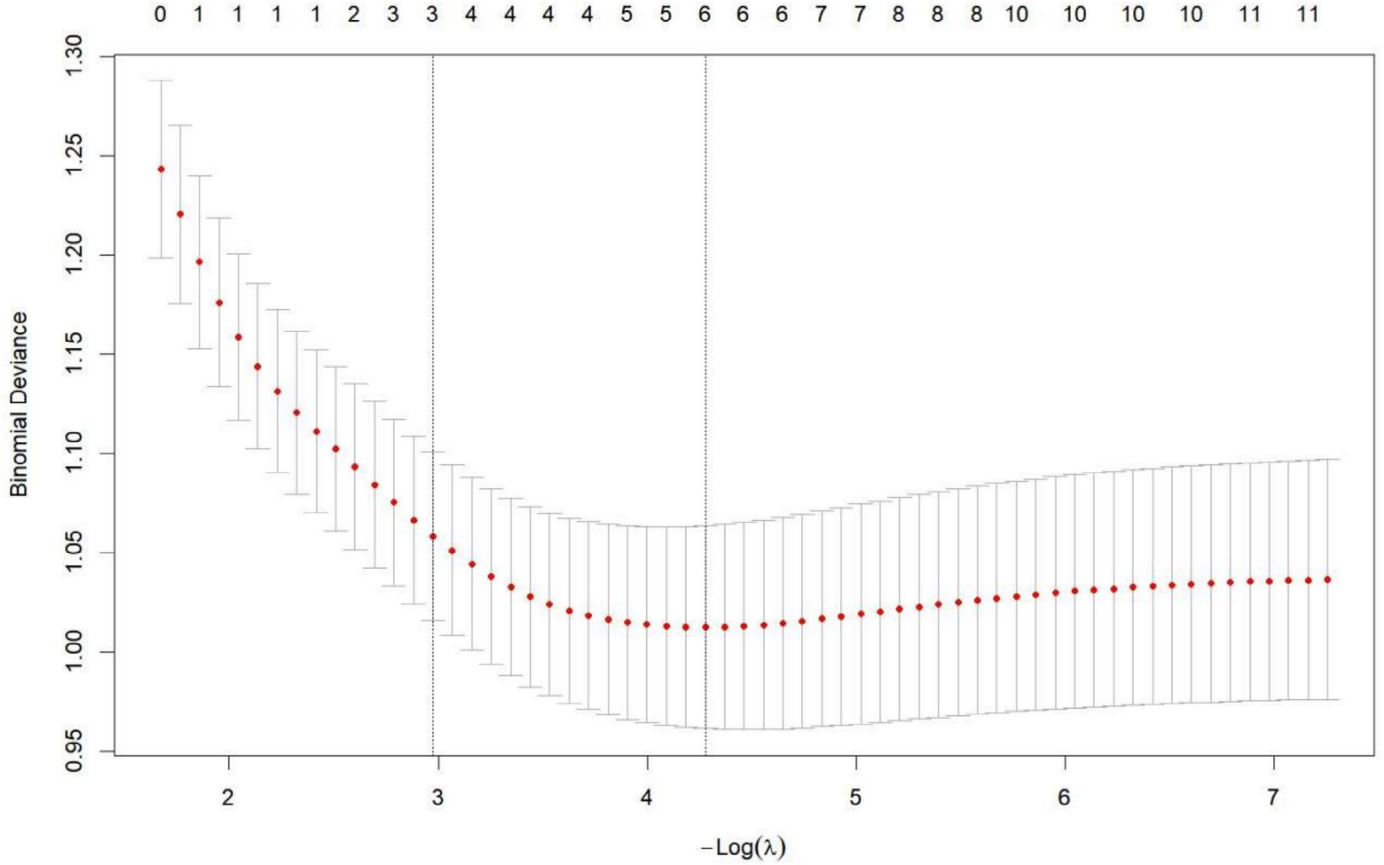
Variable selection by LASSO regression with 10‐fold cross‐validation.

**TABLE 3:** Variable coding and assignment in the logistic regression analysis.

Variable	Categories/Coding
Intrapartum blood loss	Original values
Postpartum depression	Original values
Perceived social support	Original values
Maternal sleep quality	Very satisfied, satisfied, fair, dissatisfied
Infant care ability	Very satisfied, satisfied, fair, dissatisfied
Breastfeeding ability	Proficient, partially proficient, non‐proficient
Infant feeding method	Exclusive breastfeeding (reference), mixed feeding, formula feeding

*Note:* Maternal sleep quality, infant care ability and breastfeeding ability were treated as ordinal variables. Infant feeding method was treated as a nominal variable and dummy coded using exclusive breastfeeding as the reference category.

**TABLE 4:** Multivariate logistic regression analysis of factors associated with postpartum fatigue.

Variable	*β*	Standard error	Wald *Z*	*p*	OR	95% CI
Constant	−3.757	0.584	−6.44	< 0.001		
Postpartum depression	0.260	0.034	7.69	< 0.001	1.297	1.216–1.389
Maternal sleep quality	0.428	0.136	3.14	0.0017	1.534	1.176–2.008
Breastfeeding ability	0.641	0.146	4.38	< 0.001	1.899	1.433–2.548
Intrapartum blood loss (per 100 mL)	0.350	0.140	2.58	0.0098	1.419	1.010–1.803

Abbreviations: CI, confidence interval; OR, odds ratio.

### 3.3 Development and Performance of the Nomogram

A nomogram was developed based on the final multivariable logistic regression model to facilitate individualized estimation of postpartum fatigue probability (Figure 3). The total score generated by the nomogram corresponded to the probability of postpartum fatigue. In the derivation set, the model showed good discrimination, with an AUC of 0.821 (95% CI: 0.762–0.880). In the internal validation set, the model maintained satisfactory discrimination, with an AUC of 0.801 (95% CI: 0.761–0.841; Figure 4). The optimal cut‐off value of 0.682 yielded a sensitivity of 75.2% and a specificity of 76.8% in the derivation set. When the optimal cut‐off value derived from the derivation (0.682) was applied to the internal validation set, the model achieved a sensitivity of 74.4% and a specificity of 73.5%, as shown in Table 5. In the derivation and internal validation sets, the model yielded positive predictive values of 87.8% and 86.2%, respectively and negative predictive values of 58.2% and 56.4%, respectively. Calibration was satisfactory, with calibration intercepts of 0 and −0.089, calibration slopes of 1.000 and 1.115 and Brier scores of 0.163 and 0.155, indicating good agreement between estimated and observed probabilities. The close agreement between the derivation and internal validation sets suggests good robustness within this study sample, but its generalizability to other populations and settings remains to be confirmed by external validation. The Hosmer‐Lemeshow test showed good calibration in both sets, with no evidence of poor fit in the derivation set (*χ* ^2^ = 8.949, *p* = 0.347) or the internal validation set (*χ* ^2^ = 4.422, *p* = 0.817). To further evaluate the clinical utility of the model, decision curve analysis (DCA) was performed, which demonstrated favourable net benefit across a wide range of threshold probabilities (Figure 5).

**FIGURE 3:**
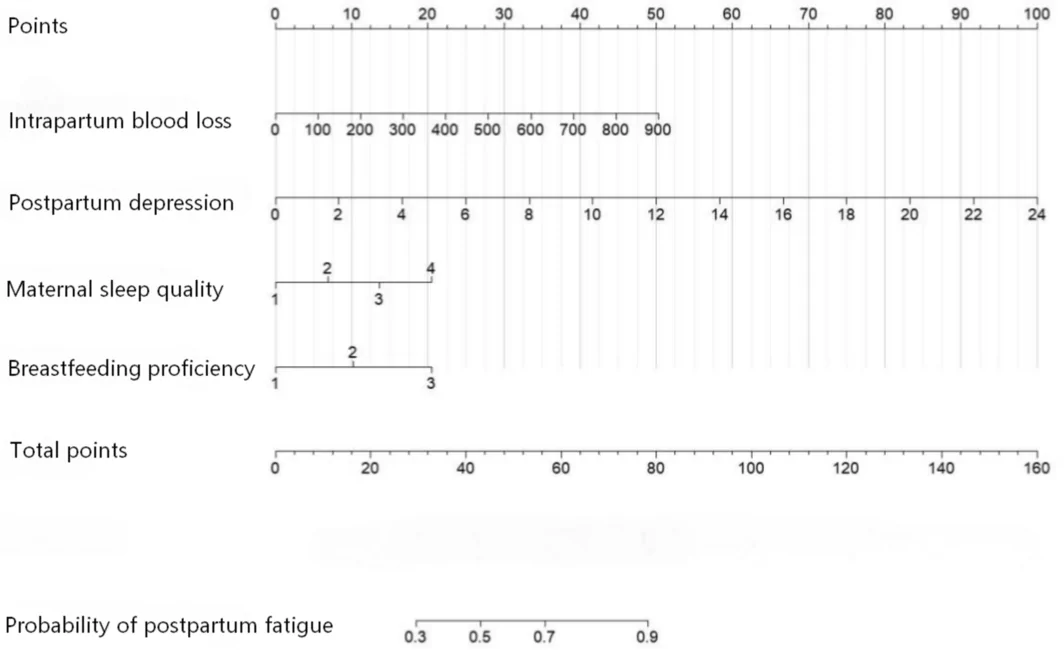
Nomogram for identifying postpartum fatigue.

**FIGURE 4:**
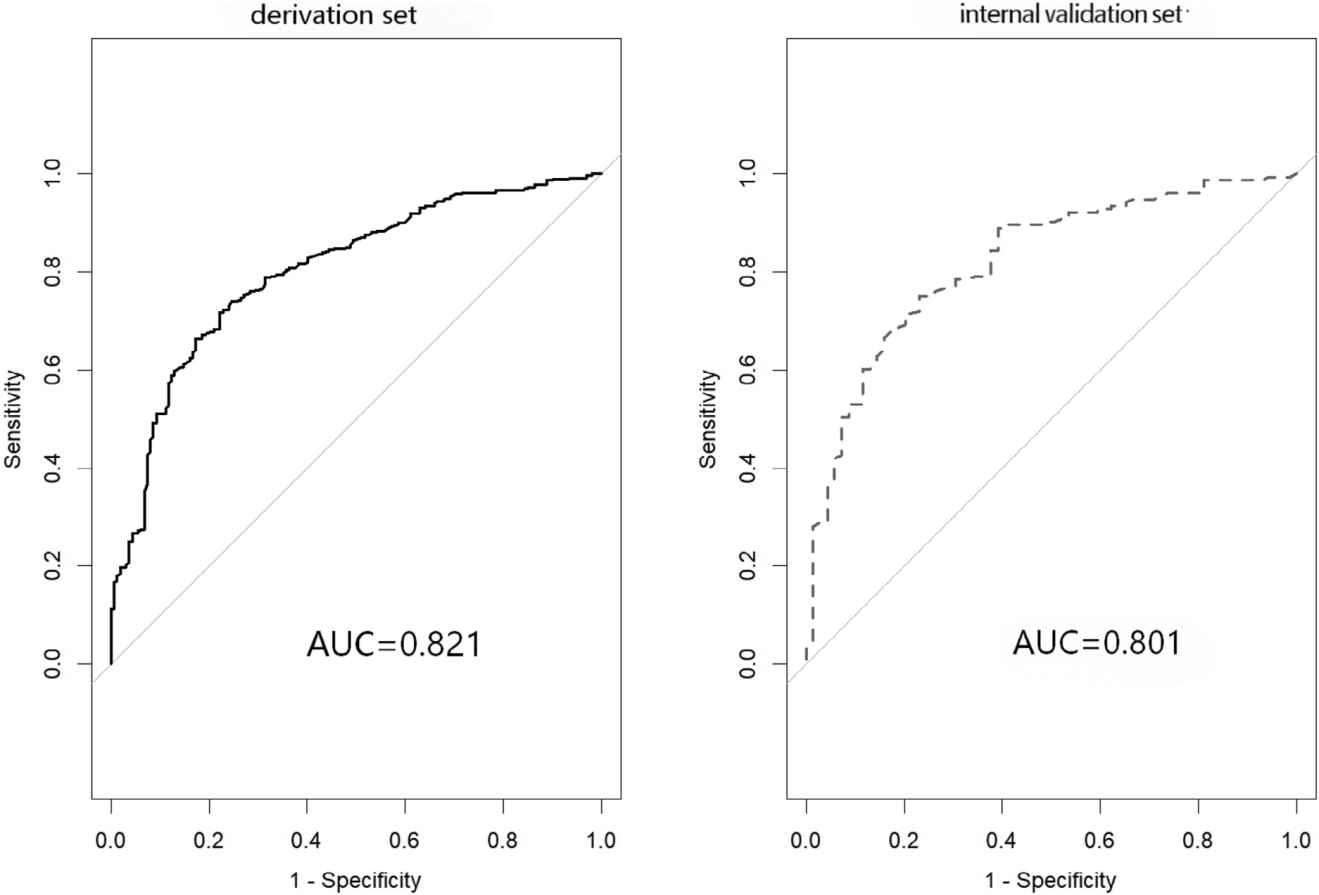
Receiver operating characteristic curves of the model.

**TABLE 5:** Model performance in the derivation and internal validation sets.

Metric	Derivation set (*n* = 522)	Internal validation set (*n* = 222)
Fatigue events, *n* (%)	360 (68.97%)	153 (68.92%)
Discrimination		
AUC (95% CI)	0.821 (0.762–0.880)	0.801 (0.761–0.841)
Sensitivity (95% CI)	0.752 (0.678–0.813)	0.744 (0.697–0.787)
Specificity (95% CI)	0.768 (0.656–0.852)	0.735 (0.662–0.797)
PPV (95% CI)	0.878 (0.811–0.923)	0.862 (0.819–0.896)
NPV (95% CI)	0.582 (0.480–0.678)	0.564 (0.497–0.629)
Calibration		
Calibration intercept	0.000	−0.089
Calibration slope	1.000	1.115
Brier score	0.163	0.155

**FIGURE 5:**
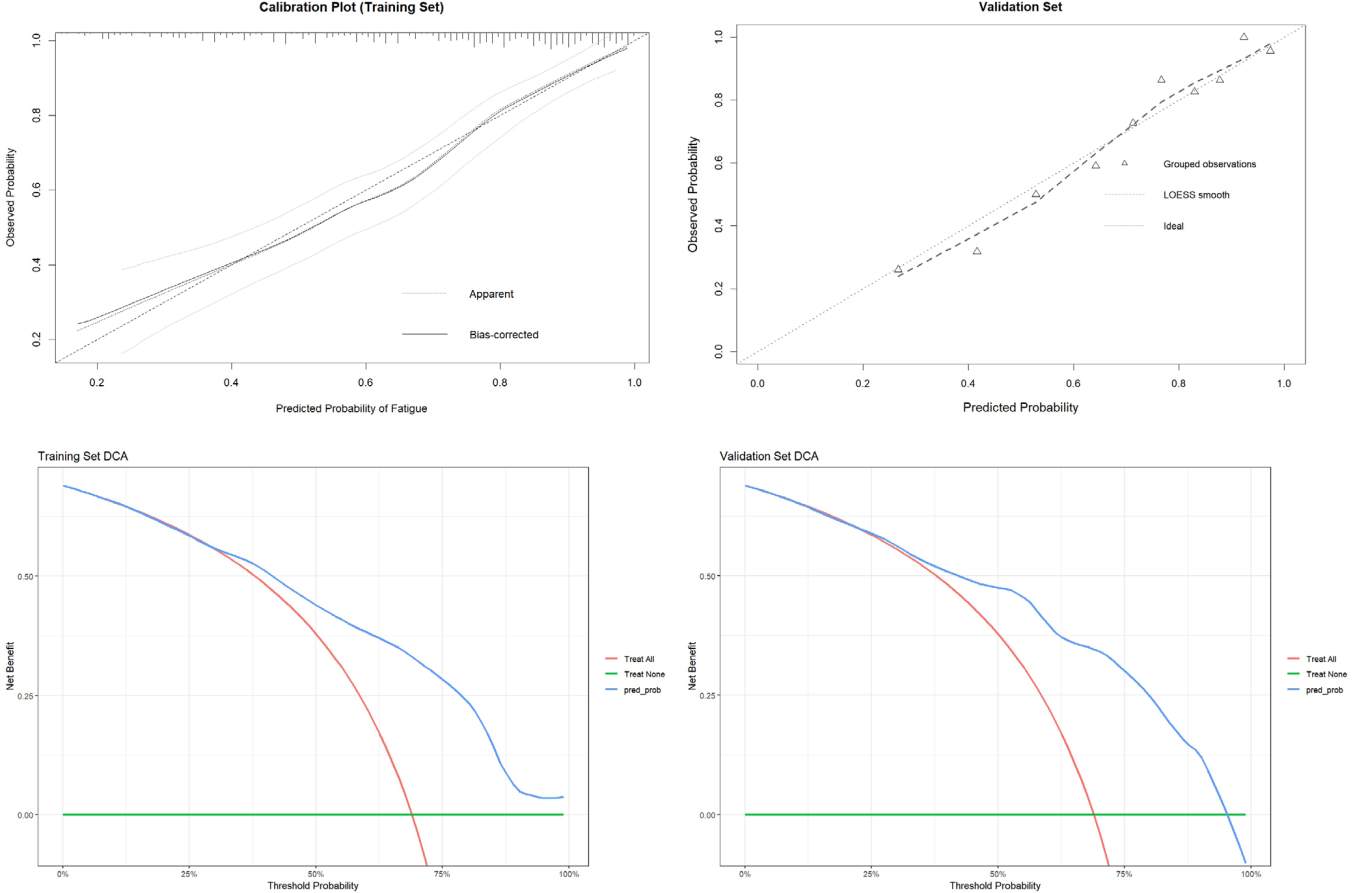
Calibration plot and decision curve analysis of the model.

## 4 Discussion

In this study, we developed and internally validated a clinical model for postpartum fatigue based on a cross‐sectional sample of postpartum women attending their routine six‐week postpartum visit. The final model included four readily available variables: postpartum depression, maternal sleep quality, breastfeeding ability and intrapartum blood loss. Although perceived social support was significantly associated with postpartum fatigue in univariate analysis, it was not retained in the final multivariable model after LASSO selection and adjustment for other factors, suggesting that its effect may be partially explained by correlated psychosocial variables such as postpartum depression and sleep quality. When applying the optimal cut‐off of 0.682 derived from the derivation set to the internal validation set, the model maintained satisfactory discriminative performance (sensitivity 74.4%, specificity 73.5%), with only a modest decline from the derivation set (75.2% and 76.8%, respectively). The nomogram showed good discrimination and calibration in both the derivation and internal validation sets, suggesting robustness within the study population and potential utility for early identification of women at higher likelihood of postpartum fatigue. However, external validation is still needed to confirm its generalizability.

### 4.1 Postpartum Depression as a Key Factor

Our findings indicate that postpartum depression was among the factors most closely associated with postpartum fatigue. This result is consistent with previous studies showing a close association between depressive symptoms and fatigue (Yi and Ahn 2024). The relationship between mood disturbance and fatigue is likely bidirectional and complex. After delivery, abrupt hormonal changes may increase vulnerability to neuroendocrine dysregulation and depressive symptoms (Yang et al. 2024). Core symptoms of postpartum depression, such as low mood and loss of interest, may also reduce the psychological reward of infant care and increase perceived emotional and cognitive burden, thereby contributing to fatigue (Wang et al. 2024). In addition, women with higher levels of postpartum depression often experience sleep disturbances such as difficulty falling asleep and early awakening. Depression‐related early awakening is not simply caused by caring for a newborn at night, but rather results from disruptions in circadian rhythms and neurotransmitters such as serotonin and norepinephrine. Depression‐related early awakening is often accompanied by feelings of helplessness and anxiety, which prevent women from falling back asleep (Ozdemir and Ozcan 2023). Therefore, routine screening for postpartum depression may help identify women at high probability of fatigue at an early stage.

### 4.2 The Foundational Role of Sleep Quality

Maternal sleep quality was another important factor associated with postpartum fatigue in our model. Consistent with prior studies (Emikpe et al. 2025), poor sleep quality appears to play a central role in the development of fatigue after delivery. Possibly due to intense infant care overnight, the postpartum period is characterized by severe fragmentation that results in a significant reduction of slow‐wave and Rapid Eye Movement (REM) sleep (Ozdemir and Ozcan 2023; Yan et al. 2022). Persistent sleep disruption may impair physical recovery, cognitive functioning and hormonal regulation, ultimately resulting in systemic fatigue. These findings suggest that healthcare providers should pay close attention to maternal sleep status during the postpartum period. Practical interventions may include sleep hygiene education, optimizing nighttime caregiving arrangements and encouraging shared infant‐care responsibilities with partners or family members.

### 4.3 Breastfeeding Ability and Maternal Energy

Breastfeeding difficulties were also independently associated with postpartum fatigue. Although breastfeeding is often considered beneficial for maternal–infant bonding and maternal role attainment (Kawashima et al. 2022; Zeng et al. 2024), difficulties such as poor latch, nipple pain, insufficient milk supply or prolonged feeding time may substantially increase physical and emotional burden. Mothers who struggle with breastfeeding may experience anxiety, self‐doubt and a sense of failure, which can further contribute to fatigue (Shen et al. 2023). These findings suggest that breastfeeding is not simply protective or harmful in itself; rather, the presence of breastfeeding difficulties may be an important source of postpartum strain. Therefore, early lactation support, individualized breastfeeding education and timely management of breastfeeding problems may help reduce maternal fatigue (Ataş and Özerdoğan 2022; Mariman et al. 2024).

### 4.4 Intrapartum Blood Loss as a Physiological Substrate

Intrapartum blood loss was another important factor of postpartum fatigue. This finding is consistent with previous research (Wilson et al. 2019) and may be explained by the physiological consequences of blood loss and postpartum anaemia. Postpartum haemorrhage is a major cause of anaemia in the postpartum period, and anaemia may affect a substantial proportion of women after delivery (Butwick and Mcdonnell 2021). Reduced haemoglobin levels can impair oxygen delivery to tissues, leading to symptoms such as lethargy, dizziness, weakness and reduced energy (Jensen et al. 2024). From this perspective, postpartum fatigue is not solely a psychological or social issue but also has a clear physiological basis. Therefore, accurate assessment of blood loss, early identification of anaemia and timely interventions such as nutritional counselling or iron supplementation may be important for preventing or alleviating postpartum fatigue.

### 4.5 Nursing Implications

The main contribution of this study is the development of a practical nomogram that translates complex statistical relationships into a clinically useful tool. This nomogram may help healthcare providers screen for postpartum fatigue probability during routine postpartum visits and may also support women in self‐assessing their own probability. In clinical practice, this tool may facilitate the early identification of women at higher probability and support timely, targeted interventions to reduce the adverse effects of postpartum fatigue and improve maternal health and well‐being.

### 4.6 Limitations

Despite its potential clinical value, several limitations should be acknowledged. First, the cross‐sectional design precludes causal inference; therefore, the observed associations should be interpreted as correlations rather than causal relationships. Second, because both the associated factors and the outcome were assessed simultaneously using self‐report questionnaires, common‐method bias may have inflated the observed associations between variables. Although standardized data collection procedures were used, this source of bias cannot be completely eliminated in a cross‐sectional survey design. Third, the use of convenience sampling and the lack of external validation may limit the generalizability and external applicability of the nomogram, thereby restricting the extrapolation of our findings to other populations and clinical settings. Future studies should conduct longitudinal and multicentre investigations, include larger and more diverse samples and perform external validation to further confirm the robustness and applicability of the model in different clinical contexts.

## 5 Conclusions

Overall, the study provides an accurate and practical model for postpartum fatigue. The nomogram integrates the major psychological, behavioural and physiological factors identified in this study and may assist in the early identification of postpartum women at high probability. If applied in clinical practice, this tool may facilitate timely, targeted interventions to reduce the adverse effects of postpartum fatigue and improve maternal health and well‐being.

## Author Contributions

**Zhidong Guo:** conceptualization, writing – review and editing, writing – original draft, methodology, investigation, formal analysis, data curation, software, validation. **Min Xu:** methodology, data curation, validation, investigation. **Shouzhen Cheng:** visualization, resources, supervision, funding acquisition.

## Funding

The authors have nothing to report.

## Ethics Statement

This study was approved by the Institutional Review Board of the First Affiliated Hospital of Sun Yat‐sen University (No. [2024]042). All participants provided written informed consent before enrollment. The study was conducted in accordance with the Declaration of Helsinki.

## Consent

All data has been anonymized, and all participants have completed a paper‐based informed consent form.

## Conflicts of Interest

The authors declare no conflicts of interest.

## Data Availability

The processed data required to reproduce the findings of this paper are available from the corresponding author upon reasonable request. The raw data are not publicly available due to their inclusion in a larger, ongoing longitudinal study. The analysis code is available from the corresponding author upon reasonable request.
